# Effect of nanostructuration on the spin crossover transition in crystalline ultrathin films†
†Electronic supplementary information (ESI) available: Materials and methods, supplementary figures and tables. See DOI: 10.1039/c8sc04935a


**DOI:** 10.1039/c8sc04935a

**Published:** 2019-02-21

**Authors:** Víctor Rubio-Giménez, Carlos Bartual-Murgui, Marta Galbiati, Alejandro Núñez-López, Javier Castells-Gil, Benoit Quinard, Pierre Seneor, Edwige Otero, Philippe Ohresser, Andrés Cantarero, Eugenio Coronado, José Antonio Real, Richard Mattana, Sergio Tatay, Carlos Martí-Gastaldo

**Affiliations:** a Instituto de Ciencia Molecular , Universitat de València , Catedrático José Beltrán 2 , 46980 Paterna , Spain . Email: eugenio.coronado@uv.es ; Email: sergio.tatay@uv.es ; Email: carlos.marti@uv.es; b Unité Mixte de Physique , CNRS , Thales , University Paris Sud , Université Paris-Saclay , 91767 Palaiseau , France; c Synchrotron SOLEIL , L'Orme des Merisiers , 91190 Saint Aubin , France

## Abstract

Mastering the nanostructuration of molecular materials onto solid surfaces and understanding how this process affects their properties are of utmost importance for their integration into solid-state electronic devices. This is even more important for spin crossover (SCO) systems, in which the spin transition is extremely sensitive to size reduction effects. These bi-stable materials have great potential for the development of nanotechnological applications provided their intrinsic properties can be successfully implemented in nanometric films, amenable to the fabrication of functional nanodevices. Here we report the fabrication of crystalline ultrathin films (<1–43 nm) of two-dimensional Hofmann-type coordination polymers by using an improved layer-by-layer strategy and a close examination of their SCO properties at the nanoscale. X-ray absorption spectroscopy data in combination with extensive atomic force microscopy analysis reveal critical dependence of the SCO transition on the number of layers and the microstructure of the films. This originates from the formation of segregated nanocrystals in early stages of the growth process that coalesce into a continuous film with an increasing number of growth cycles for an overall behaviour reminiscent of the bulk. As a result, the completeness of the high spin/low spin transition is dramatically hindered for films of less than 15 layers revealing serious limitations to the ultimate thickness that might be representative of the performance of the bulk when processing SCO materials as ultrathin films. This unprecedented exploration of the particularities of the growth of SCO thin films at the nanoscale should encourage researchers to put a spotlight on these issues when contemplating their integration into devices.

## Abstract 2

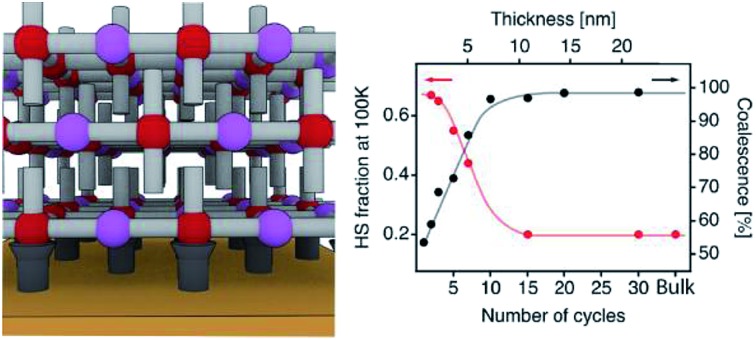
Film thickness and microstructure critically affect the spin crossover transition of a 2D coordination polymer.

## Introduction

The integration of Fe^II^ spin crossover (SCO) materials into electronic devices has attracted substantial attention in recent years.^1^–^5^ This interest resides in implementing devices with reversible switching of the electronic configuration of the SCO Fe^II^ centers between the diamagnetic low-spin (LS) and paramagnetic high-spin (HS) states. This switching can be triggered by using a variety of external inputs like temperature, light, pressure, analytes and magnetic or electric fields.^6^–^8^ Interestingly, some of these systems also exhibit a remarkable memory effect in the solid state as a result of the strong cooperative elastic interactions that operate between metal centers.^9^–^12^ This has been successfully exploited from a technological point of view with the integration of these compounds into molecular-based memory devices, which can be downscaled down to the nanometric scale using SCO nanoparticles and single molecules.^13^–^16^ Notwithstanding these remarkable achievements, practical use of these devices has been seriously hampered by the low reproducibility of device performance and low stability. This is arguably linked to the synthetic limitations imposed by the processing of SCO materials as ultrathin films with exquisite control over their thickness, morphology, crystallinity, roughness and orientation, all to play a key role in controlling device performance and ensuring a reproducible response. In fact, controlled deposition and structuration of SCO materials onto surfaces constitutes a key step in the fabrication of electronic devices and still remains a scientific and technological challenge.^8^,^17^,^18^ One of the proposed routes is the vacuum deposition of sublimable SCO complexes. This method was first introduced by Shi *et al.*^19^ and allows production of high-quality films in the sub-monolayer to sub-micrometric range with precise control of thickness. Unfortunately, this method requires expensive equipment and is limited to complexes that are stable towards decomposition in order to avoid fragmentation upon deposition.^20^ Moreover, sublimation typically leads to a decrease or even complete disappearance of the crystallinity of the deposited material, which in combination with interactions with the substrate, can strongly affect the SCO properties of the system.^20^–^27^ Thus far only a handful of sublimable SCO complexes have been used to produce thin films featuring magnetic hysteresis. However, even when these films are relatively thick (>100 nm), they generally display more gradual spin transitions than the corresponding bulk crystalline materials, suggesting a lower degree of cooperativity in the nanostructured materials.^25^,^28^–^30^ This is arguably an intrinsic limitation to this approach, as the same intermolecular interactions required for endowing the solid with long-range cooperativity are detrimental to the ability of the complexes to be sublimated, which generally results in the formation of amorphous films; nevertheless crystalline films have been obtained *via* post-synthetic annealing.^25^,^31^

The assembly of crystalline SCO frameworks onto solid substrates directly from their molecular components by using a bottom-up approach is an alternative route that might contribute to filling this gap. Although the choice of materials is also limited to 2D or 3D coordination polymers, providing exquisite control over the fabrication of the films, this methodology might allow translation of the cooperative bi-stability exhibited by bulk materials to nanostructured solid devices. We have recently demonstrated that Langmuir–Blodgett (LB) and layer-by-layer (LbL) sequential deposition, coupled with the use of substrates functionalized with self-assembled monolayers (SAMs), enables the production of crystalline, ultrathin films of metal–organic frameworks (MOFs) that retain the porosity and the electrical conductivity for thicknesses below 10 nm.^32^,^33^ This approach is better suited to process layered solids with weak interactions between neighbouring layers so that their structure can be replicated by sequential transfer of their constituent layers, formed by pre-compression in the LB trough at the liquid–liquid interphase. Moreover, this approach enables fine control of the thickness of the film simply with the number of LbL deposition cycles, an ideal scenario to investigate the effect of nanostructuration on SCO cooperativity in different stages of the growth of crystalline ultrathin films. Previous studies of nanoparticles demonstrate that there is a close relationship between their size and the critical temperature, and the completeness and hysteresis width of the SCO transition in most of the materials investigated so far.^34^–^36^ However, this information remains unclear for SCO films with nanometric thicknesses, even though they are arguably more adequate for their integration as active channels into operational devices based on the higher surface area offered by their 2D geometry for a better coupling with the bottom electrode. Direct evaluation of the films' microstructure and critical film thickness, which is required for preserving SCO properties similar to those exhibited by the bulk phase, is a crucial step prior to their integration as active materials into addressable electronic devices.

Here, we use a bottom-up approach to fabricate ultrathin films of [Fe(py)_2_{Pt(CN)_4_}] (py = pyridine) with controllable thickness, a well-known SCO layered Hofmann-type coordination polymer (HCP).^37^ First, we optimize the fabrication protocol to make it sequential (transfer of one single cell per deposition cycle), adaptable to automated cycling for higher efficiency and reproducibility, and amenable to multiple substrates. We have characterized the films by using infrared reflection absorption (IRRAS), X-ray photoelectron spectroscopy (XPS), atomic force microscopy (AFM) and two-dimensional grazing incidence X-ray diffraction (2D-GIXRD). Detailed characterization of the chemical nature of the films, their crystallinity and the evolution of their thickness and microstructure upon sequential growth is key to validate our experimental findings. The effect of nanostructuration on SCO bi-stability is directly studied by correlating AFM and X-ray absorption spectroscopy (XAS) data for films with variable thickness ranging from 40 down to 0.7 nm (30 to 1 layer). Our XAS results reveal that a SCO transition reminiscent of the bulk phase can be only detected for films with thicknesses of at least 12 nm (∼15 layers). Thinner films display a steep increase in the HS fraction at low temperature as their thickness is downsized to values corresponding to that of a single cell. Overall, our results suggest that nanosizing below a critical limit can have a detrimental effect on the properties of SCO ultrathin films as a result of the evolution of the film microstructure from coalesced to segregated nanocrystals.

## Results and discussion

### Bottom-up fabrication of ultrathin films of Fe^II^ Hofmann-type coordination polymers (Fe^II^-HCPs)

**Fig. 1:**
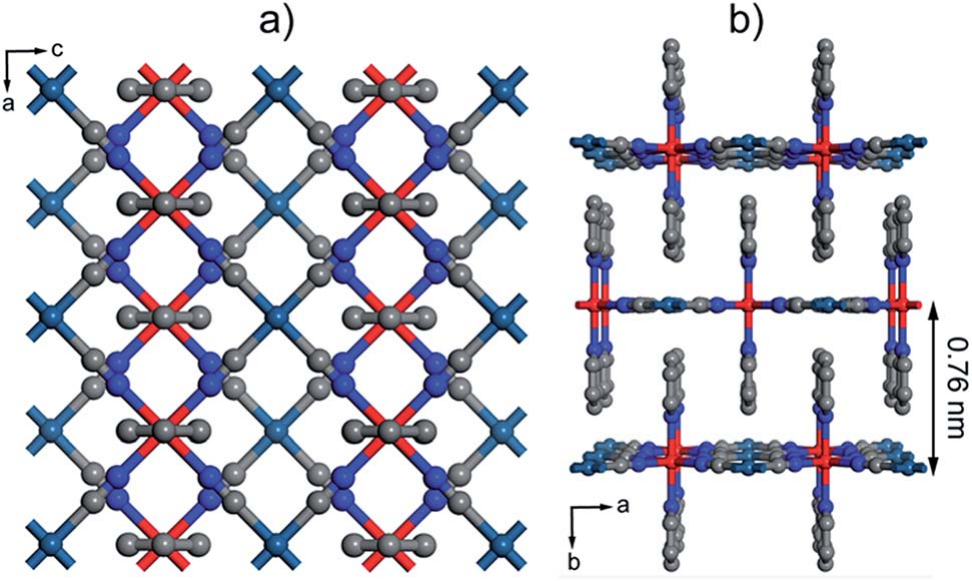
Structure of [Fe(py)_2_{Pt(CN)_4_}]. (a) Perspective along the *b* axis showing the structure of the grid-like layers. (b) Packing is controlled by π–π interactions between neighbouring layers. The thickness of a single layer corresponds to half the length of the *b* axis, 0.76 nm. Colour code: Fe, red; Pt, turquoise; N, blue; C, grey.

Within the wide variety of 2D and 3D SCO materials reported,^38^,^39^ Fe^II^-HCPs are one of the most extensively investigated ones.^39^ Their structure is composed of grid-like cyanide–metal layers interconnected by nitrogen-based ligands (Fig. 1a). The denticity of these organic linkers determines the dimensionality of the network to be 2D or 3D. These layers alternate octahedral Fe^II^ centers, responsible for the SCO phenomenon, and divalent group 10 metal centers with a square planar geometry. The Fe^II^ atoms are coordinated to the N of four cyanide groups and also to two additional pillaring ligands. If they are bis-monodentate, the resulting structure is generally 3D as a single axial ligand covalently bonds two Fe^II^ of adjacent layers. On the other hand, the use of monodentate ligands (usually py derivatives) yields a 2D structure with weak π–π interactions interconnecting neighbouring layers (Fig. 1b). There are previous reports that confirm the processability of this family of SCO materials into thin films by LbL growth, which was originally conceived by Mallouk.^40^ Afterwards, Bousseksou and co-workers used this procedure to grow several 3D Fe^II^-HCPs.^13^,^41^–^44^ These early experiments suggest that the fast ligand exchange rate of Fe^II^ ions is a crucial factor to control the LbL growth of 3D systems. Thus the sequential assembly cycles had to be performed at very low temperatures, at least –60 °C,^45^ in order to avoid desorption of the coordinated Fe^II^ ions when washing away the excess salt. Recently, Kitagawa *et al.* succeeded in producing 2D Fe^II^-HCP thin films at room temperature by putting an excess amount of an axial ligand in Fe^II^ and [Pt(CN)_4_]^2–^ salt solutions instead of in a separate one.^46^,^47^ This approach was originally used to study the effects of dimensionality on the structural transformations that are induced by loading different guests into this family of materials. Although 3D Fe^II^-HCPs have showed a lower dependence of the SCO transition with particle size reduction,^48^ we decided to adapt the methodology for 2D Fe^II^-HCPs because it offers important technical advantages that could help address the synthetic problems intrinsic to the fabrication of Fe^II^ SCO films. First, it is not necessary to keep the solutions cold using inconvenient acetone–dry ice or chloroform–dry ice mixtures. This has permitted us to automate the LbL procedure and the fabrication of thicker films, which require a greater number of immersion cycles, with higher efficiency and excellent reproducibility. Moreover, we carried out the fabrication process inside a glovebox using anhydrous de-oxygenated solvents. This would easily avoid the oxidation of Fe^II^ ions in solution that might contaminate the films for spurious magnetic contributions.

### Optimization of the fabrication conditions of [Fe(py)_2_{Pt(CN)_4_}] ultrathin films by sequential transfer

**Fig. 2:**
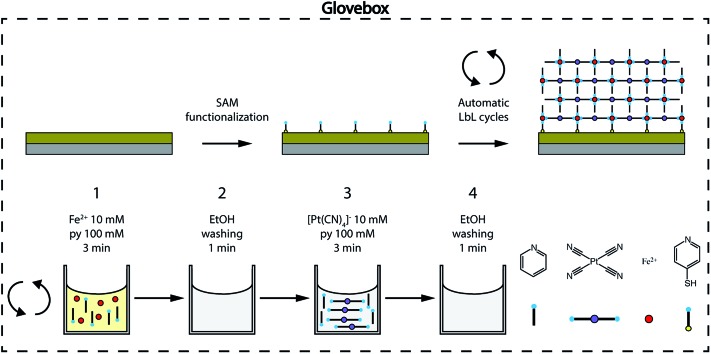
Ultrathin film fabrication. The [Fe(py)_2_{Pt(CN)_4_}] schematic structure and automated LbL fabrication process detailing four different molecular components: the axial ligand py, the [Pt(CN)_4_]^2–^ complex, the Fe^2+^ centers and the py-SH SAM. The SAM-functionalized Si/Ti/Au substrate is sequentially immersed in ethanol solutions of the molecular components, first Fe^2+^/py (1) and then [Pt(CN)_4_]^2–^/py (3) with intermediate washing steps in-between them (2 and 4). Sequential cycling for controllable film thickness is performed using an automatic dipping system to ensure reproducibility. The whole process is carried out inside a glovebox to avoid Fe^II^ oxidation.

**Fig. 3:**
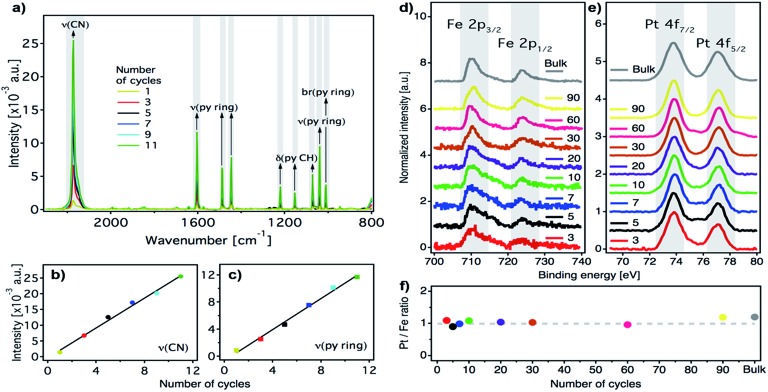
Characterization of the [Fe(py)_2_{Pt(CN)_4_}] ultrathin film as a function of the number of cycles. (a) IRRAS spectra after successive immersion cycles. Abbreviations: *ν*, stretching; *δ*, in-plane bending; br, breathing. (b) Maximum absorbance of the most intense peak, *ν*(CN) at 2173 cm^–1^*versus* the number of cycles. Data have been fitted to a linear model (black line). (c) Maximum absorbance of the most intense *ν*(py ring) peak, at 1604 cm^–1^*versus* the number of cycles. Data have been fitted to a linear model (black line). High-resolution XPS spectra for a set of films and the bulk reference showing the Fe 2p (d) and Pt 4f (e) regions. (f) The Pt/Fe ratio remains almost constant and is consistent with the theoretical value (dashed grey line) for successive immersion cycles.

We prepared ultrathin films of [Fe(py)_2_{Pt(CN)_4_}] by sequential immersion of Au substrates, previously functionalized with a 4-mercaptopyridine (py-SH) SAM, in 10 mM Fe(BF_4_)_2_/100 mM py and 10 mM (TBA)_2_Pt(CN)_4_/100 mM py ethanol solutions with intermediate ethanol washing steps (Fig. 2) under strict anaerobic conditions. We found that, when Fe(BF_4_)_2_/py solutions are prepared using anhydrous de-oxygenated ethanol under strict anaerobic conditions, they are stable, do not fade from bright yellow to brownish within a few minutes, and the appearance of a brownish precipitate indicative of the oxidation of Fe^II^ to Fe^III^ in solution is not observed (Fig. S1†). As commented above, preventing Fe^III^ contamination in the fabrication of the films is of utmost importance to ensure that their magnetic response can be exclusively associated with SCO Fe^II^ centers incorporated into the Fe^II^-HCP structure. The reproducibility of the procedure was further guaranteed by using an automated multi vessel dip coater (Fig. S2†), which ensures that the amount of the deposited material is equal from cycle to cycle. This was confirmed by the linear dependence of the intensity of the C≡N (2173 cm^–1^) and the py ring stretching (1600–1000 cm^–1^) IRRAS bands against the number of cycles (Fig. 3a–c). Both signals increase in parallel, suggesting that they are incorporated with a constant ratio into the film. Moreover, the infrared reflection absorption spectroscopy (IRRAS) spectra of the films perfectly match the FT-IR and Raman spectra of bulk [Fe(py)_2_{Pt(CN)_4_}] (Fig. S3 and S4†), which confirms the formation of the 2D Fe^II^-HCP and rules out the deposition of starting materials. Nevertheless, we further confirmed the chemical purity of our [Fe(py)_2_{Pt(CN)_4_}] ultrathin films using XPS. Survey spectra (Fig. S5†) of the film and bulk samples show the presence of the constitutive elements of [Fe(py)_2_{Pt(CN)_4_}] (Fe, Pt, C and N). High resolution Fe 2p spectra of the bulk material and the successive thin films (Fig. 3d) are comparable and display the main Fe(2p_3/2_) peak centered at 710 eV, along with a weaker Fe(2p_1/2_) peak at 723.6 eV. Both peak positions, spin–orbit splitting (13.6 eV), peak width (approximately 3.8 eV for Fe(2p_3/2_) peak) and the presence of satellites above the main lines are in consonance with those observed for other Fe^II^-HCPs and are in agreement with the presence of Fe^II^ in the HS state for both thin film and bulk materials.^49^,^50^ High resolution Pt 4f spectra (Fig. 3e), indicate the presence of the same Pt^II^ species in thin film and bulk materials, with distinctive Pt(4f_7/2_) and Pt(4f_5/2_) contributions centered at 73.8 eV and 77 eV, respectively.^49^ The Pt/Fe ratio, quantified for the whole series of ultrathin film samples and for the bulk material, remains close to the theoretical value of 1 : 1 regardless of the number of cycles (Fig. 3f), further supporting the reproducibility of the procedure.

### Crystalline ultrathin films featuring preferential orientation and controllable thickness

**Fig. 4:**
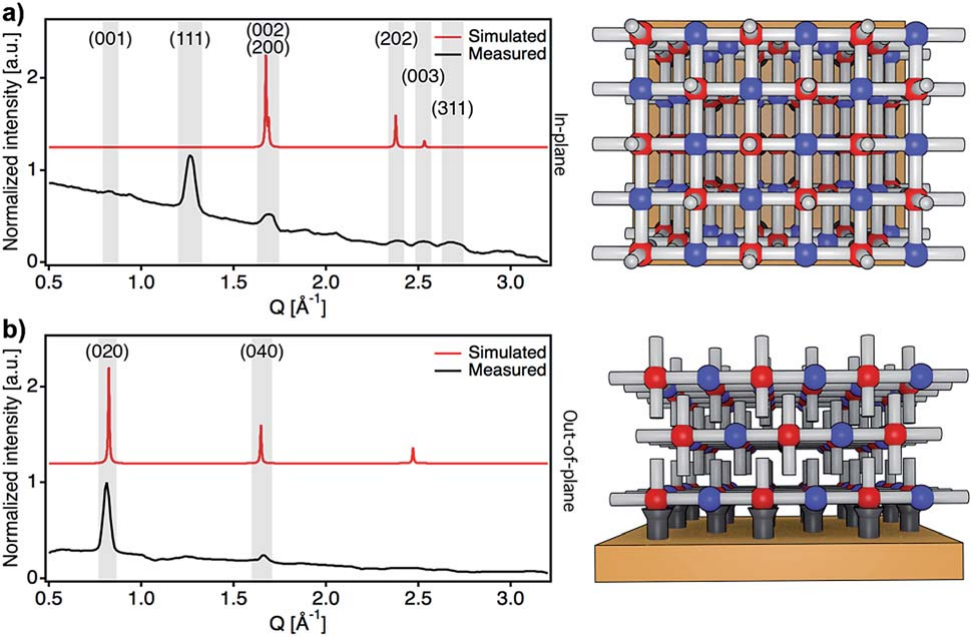
Structural characterization of films. Synchrotron in-plane (a) and out-of-plane (b) 2D-GIXRD profiles of the [Fe(py)_2_{Pt(CN)_4_}] ultrathin film (30 cycles, 22 nm thick) compared to the simulated patterns along with respective schematic diagrams showing the preferred orientation of the film with respect to the substrate.

After confirming the chemical purity of the [Fe(py)_2_{Pt(CN)_4_}] ultrathin films produced, we studied their crystallinity and orientation relative to the substrate by using synchrotron 2D-GIXRD (*λ* = 0.8533 Å, room temperature). Diffraction patterns were recorded in a single shot real-space image, which was then transformed into a Q-space image. Next, in-plane and out-of-plane profiles were extracted by simple sector integration (Fig. S6†). Fig. 4 shows in-plane and out-of-plane 2D-GIXRD patterns for a 30-cycle film (∼22 nm thick). For a clearer comparison, in-plane and out-of-plane diffraction patterns simulated from the single-crystal X-ray previously reported are also presented.^46^ The out-of-plane signal matches perfectly the simulated one, with peaks [020] and [040] clearly visible. For the in-plane geometry, all the simulated most intense peaks are visible in the experimental diffraction pattern along with the [111] and [311] peaks, which is due to the slight tilt of the [Fe{Pt(CN)_4_}] layers with respect to the substrate plane.^46^ The drastic change in the intensity of the [0*k*0] peaks, linked to the periodicity of the framework along the *b* axis, for the two geometries also suggests that the [Fe{Pt(CN)_4_}] planes adopt a disposition almost parallel with respect to the Au substrate with the py linkers pointing outwards and almost perpendicular to the solid support.

**Fig. 5:**
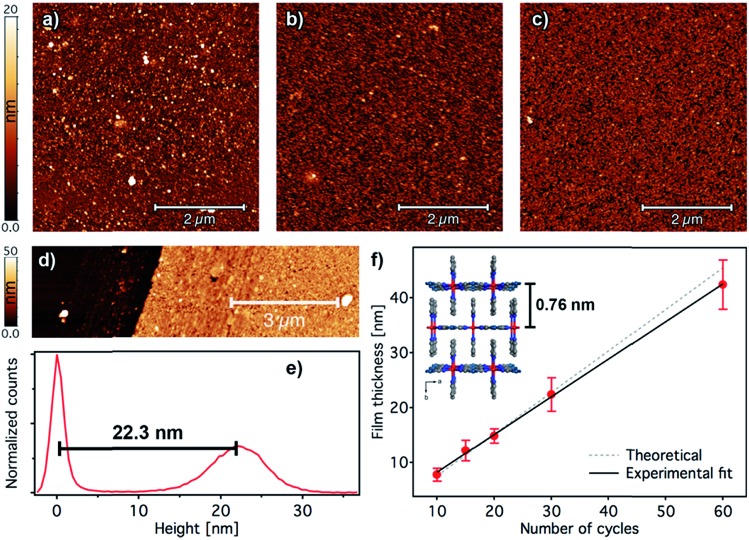
Surface morphology and thickness analysis of [Fe(py)_2_{Pt(CN)_4_}] ultrathin films. 5 × 5 μm^2^ AFM topography images of 2 (a), 5 (b), and 10 (c) cycle samples. (d) Example of an AFM topographic image of a manually scratched 30-cycle film and its corresponding height distribution (e) showing a thickness of 22.3 nm. (f) Evolution of the film thickness with the number of cycles. Height distributions were extracted from AFM images of manually scratched samples. Experimental thickness values were calculated by averaging at least three images of at least two samples; error bars correspond to standard deviations. A fit of the film thickness data *versus* the number of cycles (black line) yields an average thickness of 0.72 ± 0.03 nm per immersion cycle. This experimental value is in excellent agreement with the theoretical value of 0.76 nm increase per layer of [Fe(py)_2_{Pt(CN)_4_}] (dashed grey line) as extracted from the reported crystal structure in the HS.^46^

The overall surface morphology, homogeneity, roughness and thickness of the films, for a qualitative assessment of their quality, were next evaluated with AFM. Topography images of the film show an even distribution of the [Fe(py)_2_{Pt(CN)_4_}] over the py-SH SAM functionalized substrate for any of the 5 × 5 μm^2^ regions analysed (Fig. 5a–c). AFM images confirm complete coverage of the substrate across micrometric areas. Next, for clear evidence of the controllable thickness formation of the films, we examined the correlation between the increase in thickness with the number of growth cycles. We manually scratched the [Fe(py)_2_{Pt(CN)_4_}] film by using a soft pointy tool and then measured the height difference between the exposed substrate and the remaining film (see Fig. 5d and e) in at least five regions of each sample for a set of films after 10, 15, 20, 30 and 60 cycles. The experimental data show a linear growth of the film thickness with the number of cycles (Fig. 5f). These data along with IRRAS data displayed in Fig. 3b and c confirm the sequential incorporation of the material into the film upon sequential cycling. The slope of the curve of 0.72 ± 0.03 nm per cycle is in excellent agreement with the theoretical layer thickness extracted from the crystal structure of [Fe(py)_2_{Pt(CN)_4_}] (half of the *b* parameter, 0.76 nm), suggesting that film formation proceeds by sequential transfer of a single crystallographic cell per cycle.

### SCO transition in [Fe(py)_2_{Pt(CN)_4_}] ultrathin films: magnetic and optical measurements

We first attempted to prove that the SCO transition intrinsic to the bulk was also present in the films by using Raman spectroscopy. Changes in the position of the Raman modes at low-temperature have been used to account for the occurrence of SCO transitions in Fe^II^-HCPs.^41^,^43^,^44^,^51^ Fig. S7† shows the Raman spectra of a 90-cycle film recorded at 300 and 79 K. As expected, C≡N and py stretching modes display a shift close to Δ*ν* = 7.5 cm^–1^ upon cooling indicative of the SCO transition.^41^,^43^,^52^ This is also consistent with the appearance of py bending and stretching modes at 1225 cm^–1^ and 1610 cm^–1^ only in the low temperature spectrum, due to the acute increase in the intensity linked to the LS state.^43^,^44^,^52^ Unfortunately, our experimental setup did not provide a sufficiently good signal-to-noise ratio to resolve the Raman spectra of a 60-cycle film. Previous Raman studies have highlighted the limitations of this technique to probe SCO transitions in films below 50 nm thickness.^41^,^43^,^44^,^51^ At this point, we considered using SQUID magnetometry; thus we fabricated and measured [Fe(py)_2_{Pt(CN)_4_}] films onto Mylar/Au substrates. As shown in Fig. S8,† for a 60-cycle film (42 nm thick, red line), we clearly observe a SCO transition upon cooling to *T*_1/2_ = 206 K with a hysteresis close to 21 K. However, equivalent measurements of a 20 nm thick film (blue line) did not provide clear evidence of magnetic bi-stability. This is likely due to the diamagnetic contribution of the Mylar substrate to the film that seriously limits the sensitivity of this technique for probing magnetic changes for films below 40 nm thickness. Therefore, as the detection limit of standard methodologies proved insufficient to study the SCO behaviour of ultrathin films, we turned to a surface-sensitive method and used synchrotron XAS spectroscopy at the Fe L_2,3_ absorption edge. By using this technique, Fe^II^ HS and LS states can be directly probed as a function of the intensity of the excitation from the Fe 2p core levels to the split unoccupied d orbitals, thus allowing for direct probing of the spin state of iron centers. Since the crystal field and orbital occupation are different for the HS and LS, the shape of the XAS spectra will change with the spin transition.

**Fig. 6:**
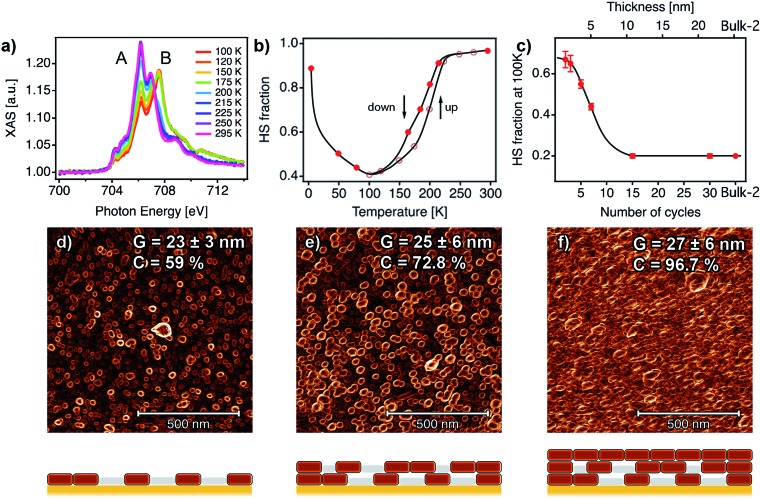
SCO behaviour in ultrathin films analysed with XAS data and correlation with the film microstructure. (a) XAS spectra as a function of temperature for a 7-cycle [Fe(py)_2_{Pt(CN)_4_}] thin film. (b) HS fraction as a function of the temperature for the 7-cycle film. (c) HS fraction at 100 K as a function of the number of cycles (1 cycle ≈ 0.72 nm). Black lines are a guide to the eye. 1 × 1 μm^2^ AFM topography images of 2 (d), 5 (e) and 10 (f) cycles after being processed with a Prewitt operator to highlight particle edges.^61^,^62^ The changes in the microstructure of the films with the number of cycles were monitored by analysing the mean lateral grain size (*G*) and film coalescence (*C*) in 1 × 1 μm^2^ AFM images (see Fig. S15, S16 and Table S2†). Schematic illustrations of the microstructure of each film are shown below each AFM image, showing the evolution from a film composed of isolated nanocrystals to a fully coalesced one.

XAS has been recently used to determine the spin state of mono/sub-monolayers of SCO complexes prepared by sublimation,^20^,^30^,^53^–^56^ but thickness dependence of spin transition has not been extensively studied for sub-10 nm thin films.^57^ XAS spectra of powder and film (∼1–20 nm) samples were recorded at the Fe L_2,3_ edges at different temperatures between 295 and 4 K. First, XAS data were collected for a bulk sample composed of single crystals of [Fe(py)_2_{Pt(CN)_4_}] (bulk-1, Fig. S9†). As can be observed, decreasing the temperature from 300 to 100 K leads to a modification of the spectral shape. This can be seen on both L_3_ and L_2_ edges where adecrease in the intensity of peaks A and A′ at the expense of signals B and B′ was observed. This variation is indicative of a SCO transition from the HS to the LS state.^22^,^24^,^53^,^58^,^59^ According to our SQUID data (bulk-1, Fig. S10†), the SCO transition is complete at 100 K with only 3.5% of the Fe^II^ atoms being still in the HS state. When the sample is cooled down to 4 K, the spectrum partially recovers its initial shape. This behaviour has been previously reported, and is the result of an X-ray induced LS → HS transition (*i.e.* SOXIESST effect).^21^,^60^ Next, we recorded XAS spectra at the Fe L_2,3_ edges on a 7-cycle sample (∼5 nm) as a function of the temperature in the 295–4 K range (Fig. 6a). The stability of the sample upon beam exposure is confirmed by comparing the spectra at 295 K collected at the beginning(end) of the cooling down(up) cycles (Fig. S11†). As can be seen in Fig. 6a, just like in the bulk solid, the temperature variation results in a change in the HS/LS population of the film. In order to assess the completeness of the SCO transition in terms of the HS/LS conversion, we estimated the HS fraction by fitting the XAS spectra for each temperature to a linear combination of the bulk XAS spectra at 295 and 100 K as described in the ESI.† Fitting results are summarized in Fig. 6b and S12.† As can be seen in Fig. 6b, a HS/LS transition takes place at 200 K and remarkably, with a hysteresis of approximately 15 K. Nonetheless, we determined a HS proportion close to 40% in the film at 100 K. Cooling down to lower temperatures only led to a marked SOXIESST effect. In Fig. 6c we have plotted the variation of the HS fraction at low temperature with the thickness of the film. The calculated HS fraction was extracted from 2- to 30-cycle samples measured at 100 K and following an equivalent fitting routine to that used for the 7-cycle sample (Fig. S13†). The evolution of the HS fraction at low temperature is especially dramatic when moving downwards from a 15-cycle sample. Particularly, in the case of the 3-cycle sample *ca.* 65% of the Fe^II^ centers remained in the HS state at 100 K while in the 15-cycle samples only 20% did. Overall, our XAS study seems to confirm that films featuring thicknesses below a critical value close to 10 nm display a marked limitation in the ability of Fe^II^ centers to undergo a SCO transition.

### Origin of the changes in the SCO transition: comparison with the bulk phase

Similar downshifting effects on the residual HS fraction at low temperature have been reported before for nanoparticles and nanocrystals of this family of Fe^II^-HCPs,^63^ but never for ultrathin films. To ascertain if the origin of this phenomenon could be ascribed to changes in the particle size, we prepared a set of polycrystalline samples of [Fe(py)_2_{Pt(CN)_4_}]. Slight changes in the synthetic conditions allowed us to isolate three samples with a decreasing crystal size from micro (bulk-1) to nanometric (bulk-2 and 3) dimensions. We evaluated the sample particle size and phase purity by direct observation *via* scanning electron microscopy (SEM) and by the relationship between peak broadening in the powder X-ray diffraction (PXRD) patterns and the crystallite size according to the Scherrer equation.^64^ As shown in Fig. S10,† SEM images and Scherrer analysis confirm a noticeable decrease in the particle size from 46 ± 11 μm to 62 ± 18 nm from bulk-1 to bulk-3 as confirmed by the crystallite sizes calculated with Scherrer's equation after LeBail refinements (Fig. S11 and Table S1†). Due to the unavailability of the additional synchrotron XAS beamtime, we used SQUID magnetometry to examine the evolution of the SCO transition. These SQUID data reveal a progressive decrease in the hysteresis width, from 44 to 10 K, and an increase of the residual HS fraction at low temperature, from close to 3.5% to 30%, when the particle size is reduced from micro to nano for higher surface to volume ratios (Fig. S10†). This confirms the dramatic effect of the particle size on the SCO transition for this material. To investigate if a similar effect could also be responsible for the evolution of the magnetic response in the films with a decreasing thickness we evaluated their overall surface morphology, homogeneity and roughness by using 1× 1 μm^2^ AFM images for a quantitative assessment of their microstructure (Fig. S15†). A deeper insight into the topography of the films reveals the presence of evenly distributed disk-shaped particles of approximately 26 ± 6 nm width. The gathered data displayed in Fig. S16 and Table S2† indicate that this mean lateral grain size (*G*) remains almost unchanged with the number of cycles after 1 cycle. Compared to the bulk phase, in which a change in the particle size close to 3 orders of magnitude induces an increase in the fraction of HS Fe^II^ centers at low-*T* close to 30% (Fig. S10†); this fraction abruptly increases from 20 to 67% in the films when the number of growth cycles is below the 15-layer limit even though the lateral particle size remains almost constant. Changes in the SCO transition in the films are more likely linked to variations in the degree of coalescence in the films (*C*). Analysis of the Prewitt edge and topography images reveals that *C* increases very rapidly with the number of cycles (Fig. 6d–f, S16 and Table S2†).^61^ As shown in Fig. S16,† it evolves from very low values, indicative of partially segregated nanocrystals that result from rapid nucleation in the early stages of the film growth. If we assume that a SCO transition only occurs in these nanocrystals, the remaining material in the film shall account for the remaining HS fraction at low temperature. In the present case, this segregated microstructure leads to a dominant fraction of non-transiting HS Fe^II^ species (*ca.* 65%). Upon increasing the thickness, after 10 cycles (∼7.6 nm thick), these nanocrystals fully coalesce forming a continuous film composed of densely packed particles, which only contain a minor fraction of the material in the HS state at low temperature (*ca.* 20%). These changes in the microstructure of the films are accompanied by a rapid increase of the surface roughness (RMS) from 1 to 10 cycles, followed by a much smoother increase (Fig. S17†) from that point onwards. Nevertheless, a 60-cycle film shows an RMS value of 6.6 ± 0.9 nm, which corresponds to less than 15% of the total film thickness. This microstructural change has a dramatic effect on the SCO properties of the thin films due to increased interparticle interactions in coalesced films. A similar effect has been previously observed for SCO nanoparticles embedded in silica or polymeric matrices,^65^–^67^ for which a decrease of the hysteresis width and the transition temperature together with an increase of the residual HS fraction was always observed when increasing interparticle distances. A direct comparison between the HS/LS population displayed by the bulk phases (SQUID) and the films (XAS) reveals differences between them. Films formed after 15 and 30 immersion cycles (12 and 22 nm thick), with a *G* of 26 ± 6 nm, display a constant HS fraction of 20% (Fig. 6c). This value is somewhat smaller than the 30% of Fe^II^ HS centers in bulk-3, even though it is based on slightly larger particles (62 ± 18 nm, Fig. S10†). The lower HS fraction observed in the films suggests that the coalescence of segregated nanocrystals into continuous films of densely packed ones can have a positive effect on the SCO behaviour. We believe that this is due to enhanced interparticle interactions in the fully coalesced films which are not as strong in bulk powders composed of nanocrystals of similar size ranges. We hypothesize that this is linked to the controlled growth process of the films, which results in the formation of homogeneous nanocrystals with a smaller size dispersion when compared to the bulk prepared by liquid diffusion under kinetic control, which facilitates the coalesce into continuous films.

Largely, our experiments confirm that the growth of films of [Fe(py)_2_{Pt(CN)_4_}] by bottom-up methodologies can be controlled down to ultrathin thicknesses whilst retaining or partially improving the SCO properties of the bulk phase. This paves the way for their implementation in bi-stable nanoelectronic devices. We tried to demonstrate this possibility by fabricating vertical cross junctions incorporating thick films up to 90 nm of the Fe^II^-HCP as an active channel (see ESI for details, Fig. S18†). Unfortunately, our vertical junctions were repeatedly short-circuited due to the diffusion of Au atoms from the evaporation of the top electrode through the Fe^II^-HCP layer. We are certain that these results could be improved by reducing the contact size, as for example using nanometric junctions.^68^

## Conclusions

We have optimized the fabrication of thin films of an interdigitated SCO 2D Fe^II^-HCP by sequential LbL growth under anaerobic conditions. Extensive chemical and structural characterization techniques are used to prove that this enables the formation of crystalline, preferentially oriented films by deposition of one single cell per cycle. This bottom-up methodology is used to produce a set of high-quality ultrathin films with varying thickness (<1–43 nm) for studying the effect of nanostructuration on SCO cooperativity. Synchrotron XAS data confirm that thin films of [Fe(py)_2_{Pt(CN)_4_}] display SCO transitions similar to the bulk phase upon thickness reduction down to a critical value (∼12 nm). Even though a thermal hysteresis of 15 K can still be detected in ∼5 nm thick films, downsizing results in the reduction of completeness and cooperativity of the spin transition. Our AFM analysis discards that these effects are linked to changes in the particle size. Other factors like crystal quality,^69^ particle shape,^48^ inter-particle organization,^65^ surface defects or particle orientation with respect to the substrate have been shown to have relevant effects on the SCO transition.^48^,^65^,^69^,^70^ In our case, this phenomenon seems to have originated by changes in the microstructure of the films in different growth stages controlled by the number of immersion cycles. Films over 10 layers (∼7.6 nm) are fully coalesced and display similar Fe^II^ HS fractions at 100 K than bulk samples of equal or even higher mean lateral grain sizes, thanks to increased interparticle interactions. The absence of the size reduction effect on the SCO transition for this range of thicknesses (>15 nm) had been observed by Akou *et al.* in thin films of other 3D Fe^II^-HCPs.^71^ In turn, below the 10-layer limit, the films display partial segregation into isolated nanocrystals separated by a non-crystalline matrix containing HS Fe^II^ ions. This results in a steep increase of the HS fraction at low temperature as the thickness is downsized, for a quick disappearance of the SCO hysteresis.

Overall, our work reveals serious limitations to the ultimate thickness that might be representative of the behaviour of the bulk when processing SCO materials as ultrathin films. Reducing their thickness below a critical value can have detrimental effects on the magnetic properties of the film. This phenomenon is probably intrinsic to the kinetics of nucleation and LbL growth of thin films. We are confident that this information will encourage the community of researchers currently working on the integration of SCO compounds into bi-stable electronic and spintronic nanodevices to re-examine the properties of thin films very carefully, especially when reaching the nanometric limit, as they might be a limiting factor to performance.

## Conflicts of interest

There are no conflicts to declare.

## Supplementary Material

Supplementary information
