# Correction: Effect of nanostructuration on the spin crossover transition in crystalline ultrathin films

**DOI:** 10.1039/c9sc90075f

**Published:** 2019-04-05

**Authors:** Víctor Rubio-Giménez, Carlos Bartual-Murgui, Marta Galbiati, Alejandro Núñez-López, Javier Castells-Gil, Benoit Quinard, Pierre Seneor, Edwige Otero, Philippe Ohresser, Andrés Cantarero, Eugenio Coronado, José Antonio Real, Richard Mattana, Sergio Tatay, Carlos Martí-Gastaldo

**Affiliations:** a Instituto de Ciencia Molecular , Universitat de València , Catedrático José Beltrán 2 , 46980 Paterna , Spain . Email: eugenio.coronado@uv.es; b Unité Mixte de Physique , CNRS , Thales , University Paris Sud , Université Paris-Saclay , 91767 Palaiseau , France; c Synchrotron SOLEIL , L’Orme des Merisiers , 91190 Saint Aubin , France

## Abstract

Correction for ‘Effect of nanostructuration on the spin crossover transition in crystalline ultrathin films’ by Víctor Rubio-Giménez *et al.*, *Chem. Sci.*, 2019, DOI: ; 10.1039/c8sc04935a.



## 

The authors regret that the name of one of the beamlines in the acknowledgements section of the original article was incorrectly given as “DEIMOS (BL-28)”. The correct name of the beamline is “BOREAS (BL-29)”.

The Royal Society of Chemistry apologises for these errors and any consequent inconvenience to authors and readers.

