# Supplementary material for: Effect of nanostructuration on the spin crossover transition in crystalline ultrathin films
Source: Chem Sci. 2019 Feb 21;10(14):4038–47. doi: 10.1039/c8sc04935a (PMC6460953; doi:10.1039/c8sc04935a)
Supplement: Supplementary file 1 [file SC-010-C8SC04935A-s001.pdf]

Electronic Supplementary Material (ESI) for Chemical Science.
This journal is © The Royal Society of Chemistry 2019

Effect of nanostructuration over the spin crossover transition in crystalline ultrathin films

Víctor Rubio-Giménez,[a] Carlos Bartual-Murgui,[a] Marta Galbiati,[a,] Alejandro Núñez-López,[a] Javier Castells-Gil,[a] Benoit Quinard,[b] Pierre Seneor,[b] Edwige Otero,[c] Philippe Ohresser,[c] Andrés Cantarero,[a] Eugenio Coronado,[a,*] José Antonio Real,[a] Richard Mattana,[b] Sergio Tatay [a,*] and Carlos Martí-Gastaldo [a,*]

MATERIALS AND METHODS 3

SUPPLEMENTARY FIGURES AND TABLES 6

Figure S1 $Fe(BF_4)_2$/py solutions prepared and stored under ambient conditions and using anhydrous de-oxygenated ethanol in anaerobic conditions 6

Figure S2 Images showing the automated multi vessel dip coater placed inside a N_2-filled glovebox 6

Figure S3 IRRAS spectrum of a 30-cycles Si/Ti (3 nm)/Au (150 nm)/[$Fe(py)_2\{Pt(CN)_4\}$] sample compared with the FT-IR spectrum of bulk [$Fe(py)_2\{Pt(CN)_4\}$] 7

Figure S4 Raman spectrum of a 90-cycles Si/Ti (3 nm)/Au (15 nm)/[$Fe(py)_2\{Pt(CN)_4\}$] sample compared with the Raman spectrum of bulk [$Fe(py)_2\{Pt(CN)_4\}$] 7

Figure S5 Survey XPS spectra of a 30-cycles Si/Ti (3 nm)/Au (15 nm)/[$Fe(py)_2\{Pt(CN)_4\}$] sample compared with bulk [$Fe(py)_2\{Pt(CN)_4\}$] 8

Figure S6 2D-GIXRD data processing 9

Figure S7 Raman spectra at 300 K and 79 K of a 90-cycles film onto Si/Ti (3 nm)/Au (15 nm)/[$Fe(py)_2\{Pt(CN)_4\}$] 10

Figure S8 SCO transition in [$Fe(py)_2\{Pt(CN)_4\}$] films as measured with SQUID magnetometry 10

Figure S9 XAS spectra measured for a single crystal sample (Bulk-1) and a microcrystalline powder sample (Bulk-2) at 295, 100 and 4 K 11

Figure S10 SCO behaviour of bulk [$Fe(py)_2\{Pt(CN)_4\}$] samples with different crystallinity and particle size 12

Figure S11 LeBail refinements of bulk [$Fe(py)_2\{Pt(CN)_4\}$] samples 13

Table S1 Summary of the parameters obtained from the LeBail refinements 13

Figure S12 7-cycles sample XAS spectra at 295 K collected at the beginning(end) of the cooling down(up) cycles 15

Figure S13 7-cycles sample XAS spectra at 295, 175 and 100 K and corresponding fitted spectra 15

Figure S14 XAS spectra at 100 K and corresponding fitted spectra as function of the number of cycles. 16

Figure S15 1 x 1 μm^2 AFM topography, Prewitt edge and phase images 17

Figure S16 Evolution of mean grain size and grade of coalescence with the number of cycles 18

Figure S17 Evolution of RMS roughness with the number of cycles 18

Table S2 Summary of the film microstructure parameters 13

Figure S18 Device fabrication 20

References.....20

MATERIALS AND METHODS

Materials and reagents. Iron(II) tetrafluoroborate hexahydrate ($Fe(BF_4)_2·6H_2O$) (Sigma-Aldrich, 97%), Mohr salt [$(NH_4)_2Fe(SO_4)_2·6H_2O$] (Acros Organics, 99%), anhydrous pyridine (py) (Sigma-Aldrich, ≥99%), ascorbic acid (Sigma-Aldrich, ≥99%), 4-mercaptopyridine (py-SH) (TCI, >97%), tetrabutylammonium bromide (TBABr) (Sigma-Aldrich, ≥98%) and potassium tetracyanoplatinate(II) hydrate ($K_2Pt(CN)_4)·3H_2O$) (Strem Chemicals, 95%) were used as received without further purification. Mylar was purchased from Du Pont Ibérica, S.L. Anhydrous ethanol (99.5%) was purchased from Acros Organics. Ultrapure milliQ water with a resistivity higher than 18 MΩ·cm was used when required. Other solvents (HPLC-grade) were purchased from Scharlab S.L. $(TBA)_2Pt(CN)_4$ was synthesized following a literature procedure.

Synthesis of [Fe(py)$_2${Pt(CN)$_4$}] Bulk-1 & Bulk-2. A solution of py (0.255 mmol, 20.2 mg) in 2 mL of methanol was added dropwise to an aqueous solution (2 mL) of Mohr salt $(NH_4)_2Fe(SO_4)_2·6H_2O$ (40 mg, 0.102 mmol) in the presence of a small amount of ascorbic acid under an Argon atmosphere. The resulting solution was stirred for 10 minutes and then an aqueous solution of $K_2Pt(CN)_4·3H_2O$ (38.4 mg, 0.102 mmol) was added dropwise observing the precipitation of a white solid from the first drop. After the addition was completed the mixture was stirred for 40 additional minutes. Afterwards the white solid was filtered, washed with cold water and methanol and dried under vacuum (yield ca. 78%). Both Bulk-1 & Bulk-2 microcrystalline samples were obtained following the same procedure due to small uncontrollable changes in the manual addition of the $K_2Pt(CN)_4·3H_2O$ solution.

Synthesis of [Fe(py)$_2${Pt(CN)$_4$}] Bulk-3. Single crystals were synthesized by slow liquid–liquid diffusion in a H-vessel tube. One arm of the vessel contained an aqueous solution (1 mL) of $Fe(BF_4)_2·6H_2O$ (0.09 mmol, 30 mg) and py (0.2 mmol, 16 mg). The other arm of the vessel contained an aqueous solution (1 mL) of $K_2Pt(CN)_4·3H_2O$ (0.09mmol, 40 mg). The rest of the tube (about 8 mL) was filled with a methanol:water solution (1:1 in volume), thus creating a proper interphase, and left to rest at room temperature. Colourless block-like crystals were obtained after about 2 weeks (yield ca. 40%).

Substrate preparation and SAM functionalization. Prior to evaporation of Au, silicon substrates were soaked in a freshly prepared solution of $H_2O_2/NH_4OH/H_2O$ (1:1:2) and sonicated for 10 min. This treatment was repeated three times. Next, they were rinsed with Milli-Q water, sonicated 5 min in Milli-Q water twice, and dried under a nitrogen stream. A 3 nm Ti layer followed by the Au layer were then evaporated in an Edwars Auto 500 thermal evaporator in a tungsten basket coated with Al_2O_3 placed inside a nitrogen glovebox. Base pressure was $2·10^{-6}$ mbar, and evaporation rate 0.02 nm·s^{-1}. 15 nm of Au was the preferred thickness except for infrared spectroscopy studies where 150 nm were used. For magnetic measurements Mylar was used as diamagnetic substrates, prior to evaporation they were cleaned by sonication in isopropanol for 10 min and then dried under a nitrogen stream. For SAM functionalization substrates were previously activated via O_2 plasma treatment (MiniPCFlecto, Plasma Technology), and then immersed in a 1 mM ethanol solution of py-SH for 24 hours. Next, substrates were rinsed with fresh ethanol and dried under a N_2 stream.

Infrared reflection absorption spectra (IRRAS). Measurements were performed on a VeeMax II sampling stage (Pike Technologies) placed in the sample compartment of a Nicolet 5700 Transformation-Infrared Spectrometer. All measurements were performed under nitrogen atmosphere. Each FT-IR spectrum represents the average of 512 scans with a resolution of 4 cm^{-1}. The infrared beam (incidence angle: 75°) was p-polarized by mean of a manual ZnSe polarizer, and the output signal was collected using a refrigerated mercury cadmium telluride (MTC) detector.

Variable-temperature Raman spectroscopy (VT-Raman). Measurements were performed on a Horiba T64000 Raman spectrometer. Sample was inserted into a continuous flow micro-cryostat (Cryovac) with a

base pressure of 10^{-6} mbar to perform temperature dependent measurements from room temperature to 79 K. Raman spectra were excited at 458 nm using an Ar ion laser and a beam power of 30 mW was maintained over the sample. A grating spectrograph of 1800 gr·mm^{-1} was used, the entrance slit was kept at 40 μm during measurements to obtain a spectral resolution of about 0.8 cm^{-1}. Spectra were recorded using an accumulation time of 3600 s due to the low signal intensity.

Atomic force microscopy (AFM). Measurements were performed with a Digital Instruments Veeco Nanoscope IVa microscope in tapping mode using silicon tips with a natural resonance frequency of 300 KHz and with an equivalent constant force of 40 N·m^{-1}. Scan rate was adjusted during the scanning of each image (0.1-1 Hz, 512 samples/line). Film thickness was evaluated by scratching off the deposited MOF film using a toothpick.

X-ray Photoelectron Spectroscopy (XPS). Measurements were performed ex situ at the X-ray Spectroscopy Service at the Universidad de Alicante using a K-Alpha X-ray photoelectron spectrometer system (Thermo Scientific). All spectra were collected using Al Kα radiation (1486.6 eV), monochromatized by a twin crystal monochromator, yielding a focused X-ray spot (elliptical in shape with a major axis length of 400 μm) at 3 mA·C and 12 kV. The alpha hemispherical analyser was operated in the constant energy mode with survey scan pass energies of 200 eV to measure the whole energy band and 50 eV in a narrow scan to selectively measure the particular elements. XPS data were analysed with Avantage software. A smart background function was used to approximate the experimental backgrounds. Charge compensation was achieved with the system flood gun that provides low energy electrons and low energy argon ions from a single source. Spectra were referenced using the C 1s main peak (285 eV).

Scanning Electron Microscopy (SEM). Particle morphologies and dimensions were studied with a Hitachi S-4800 scanning electron microscope at an accelerating voltage of 20 keV, over metalized samples with a mixture of gold and palladium during 30 s.

Powder x-Ray Diffraction (PXRD). Patterns were collected for polycrystalline samples using 0.5 mm glass capillaries which were mounted, aligned and measured in a PANalytical Empyrean diffractometer (Bragg-Brentano geometry) using copper radiation (Cu Kα = 1.5418 Å) with an PIXcel detector, operating at 40 mA and 45 kV. Profiles were collected by using a Soller Slit of 0.02° and a divergence slit of ¼ at room temperature in the angular range $3° < 2\theta < 50°$ with a step size of 0.013°. LeBail refinements were carried out with the FULLPROF software package.[1]

2D Grazing Incidence X-Ray Diffraction (2D-GIXRD). Synchrotron 2D-GIXRD measurements were performed at beamline I07 at Diamond synchrotron (proposal SI14495). Samples were placed into a closed cell mounted on a multiaxis diffractometer and measured under a continuous He flow. Single shot images (1 second exposition) were recorded at room temperature using a Pilatus 2M area detector with wavelength of 0.85 Å (beam energy of 14.53 keV), a sample-to-detector distance of 456 mm and an incidence angle of 0.1°. Data was analysed and processed using DAWN software.[2]

Magnetic measurements. Variable-temperature magnetic susceptibility data were recorded on a Quantum Design MPMS-XL-5 SQUID magnetometer equipped with a 7 T magnet, operating at 1 T and a temperature range of 1.8−400 K. Experimental susceptibilities were corrected for diamagnetism of the constituent atoms by the use of Pascal's constants. Mylar was used as a diamagnetic substrate for thin film measurements. In bulk samples the HS fraction at 300 K was adjusted to 100% based on previous Mössbauer spectroscopy studies[3,4] and the Fe^{II}-N distances reported in the crystal structure of $[Fe(py)_2\{Pt(CN)_4\}]$ at 298 K.[5]

X-ray absorption spectroscopy (XAS). Iron $L_{2,3}$-edge XAS measurements were performed at the DEIMOS beamline[6] at SOLEIL synchrotron facility and at the BOREAS (BL-29)[7] beamline at ALBA synchrotron using circular polarized light. Samples were irradiated with a beam spot size of 800 x 800 μm^2 in ultrahigh vacuum conditions (10^{-10} mbar) at a temperature range of 295-4 K. The beam spot was maintained at approximately the same spot for the whole study by compensating for the temperature change drift in the sample position. A magnetic field up to 1 T was applied perpendicular to the plane. The signal from the sample was detected in total electron yield (TEY) mode. The time required to acquire one spectrum was 3 min. We averaged 8 consecutive XAS spectra to obtain improve signal-to-noise ratio.

Determination of the HS fraction (γHS) in XAS spectra. The thin film sample spectra measured as a function of the temperature (I_T^{film}) were simulated between 700 and 715 eV by linear combination of the spectra measured at 300 K and 100 K of the bulk sample ($I_{300K}^{bulk}, I_{100K}^{bulk}$).

$$I_T^{film} = xI_{300K}^{bulk} + (1-x)I_{100K}^{bulk} \quad (1)$$

According to SQUID data (Figure S10) the HS fraction at 300 K and 100 K for the bulk sample 3 are 100% and 3.5%, respectively. As a result, the γ_{HS} can be written as follow:

$$\gamma_{HS}^{film} = 3.5 + 96.5 \cdot x \quad (2)$$

Before the fitting, a linear background was subtracted to all spectra and the following normalization constraint was applied:

$$\int_{700}^{715} \left(I_T^{film} - 1\right) dE = 1 \quad (3)$$

SUPPLEMENTARY FIGURES AND TABLES

Figure S1. Images showing $Fe(BF_4)_2$/py solutions prepared and stored under ambient conditions and using anhydrous de-oxygenated ethanol in anaerobic conditions. Under ambient conditions, the solution turns dark brown at first and then, after 3 hours the appearance of a small amount of precipitate is visible, along with the lightening of the solution.

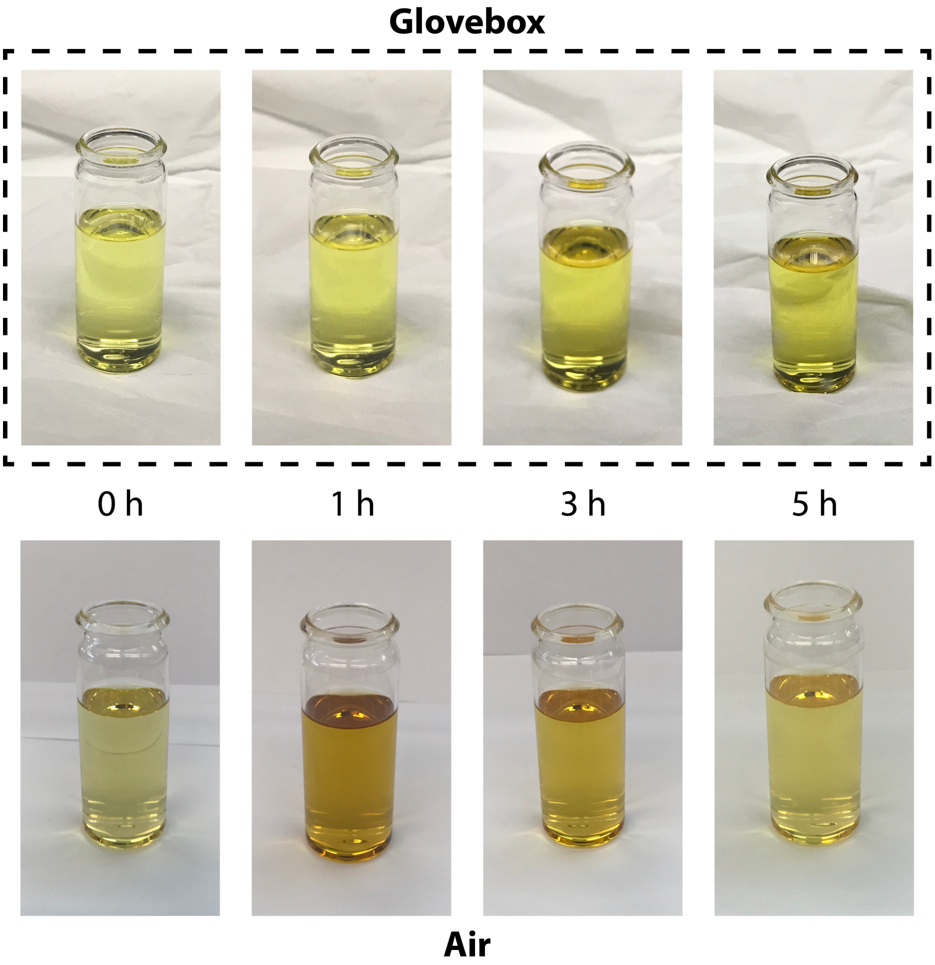

Figure S2. Images showing the automated multi vessel dip coater placed inside a N_2-filled glovebox.

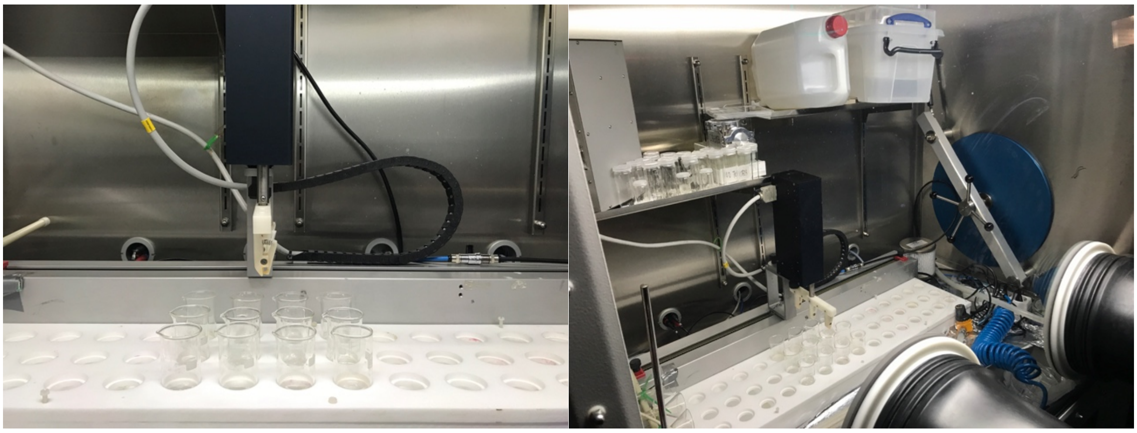

Figure S3. IRRAS spectrum of a 30-cycles Si/Ti (3 nm)/Au (150 nm)/[Fe(py)$_2${Pt(CN)$_4$}] sample (green) compared with the FT-IR spectrum of bulk [Fe(py)$_2${Pt(CN)$_4$}] (red). Assigned vibrations are highlighted in grey.

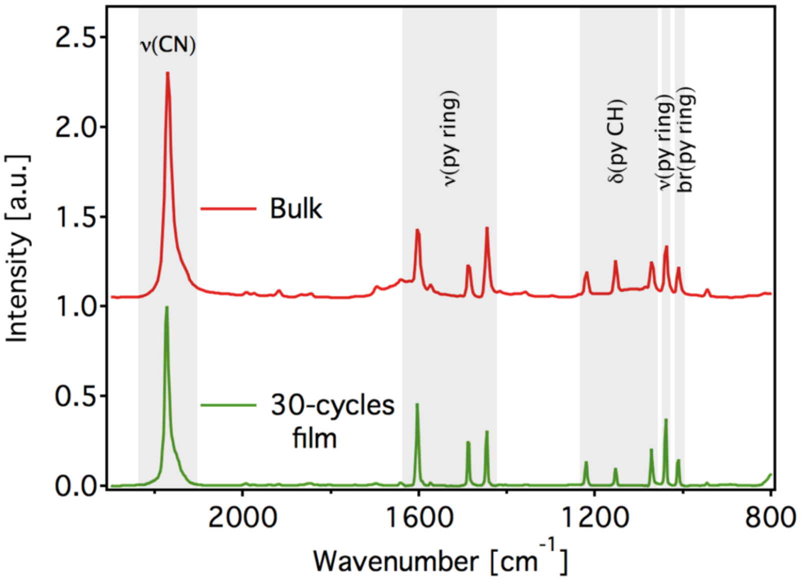

Figure S4. Raman spectrum of a 90-cycles Si/Ti (3 nm)/Au (15 nm)/[Fe(py)$_2${Pt(CN)$_4$}] sample (black) compared with the Raman spectrum of bulk [Fe(py)$_2${Pt(CN)$_4$}] (red). Assigned vibrations are highlighted in grey. Peak assigned with (*) was found to be characteristic of the film deterioration due to the long exposition time required to record spectra with acceptable signal-to-noise ratio. A laser wavelength of 458 nm was used to record the 90-cycles film spectrum and a laser wavelength of 638 nm was used to record the bulk spectrum.

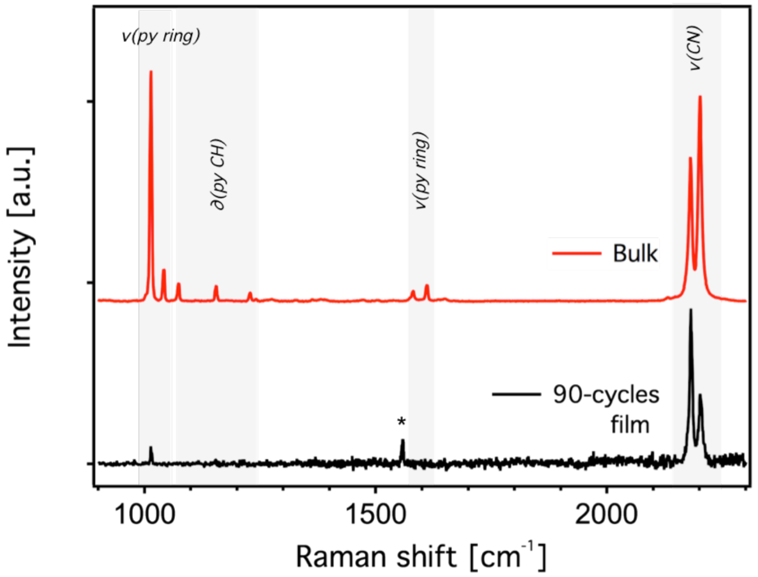

Figure S5. Survey XPS spectra of a 30-cycles Si/Ti (3 nm)/Au (15 nm)/[Fe(py)$_2${Pt(CN)$_4$}] sample (green) compared with bulk [Fe(py)$_2${Pt(CN)$_4$}] (red). Assigned peaks are highlighted in grey.

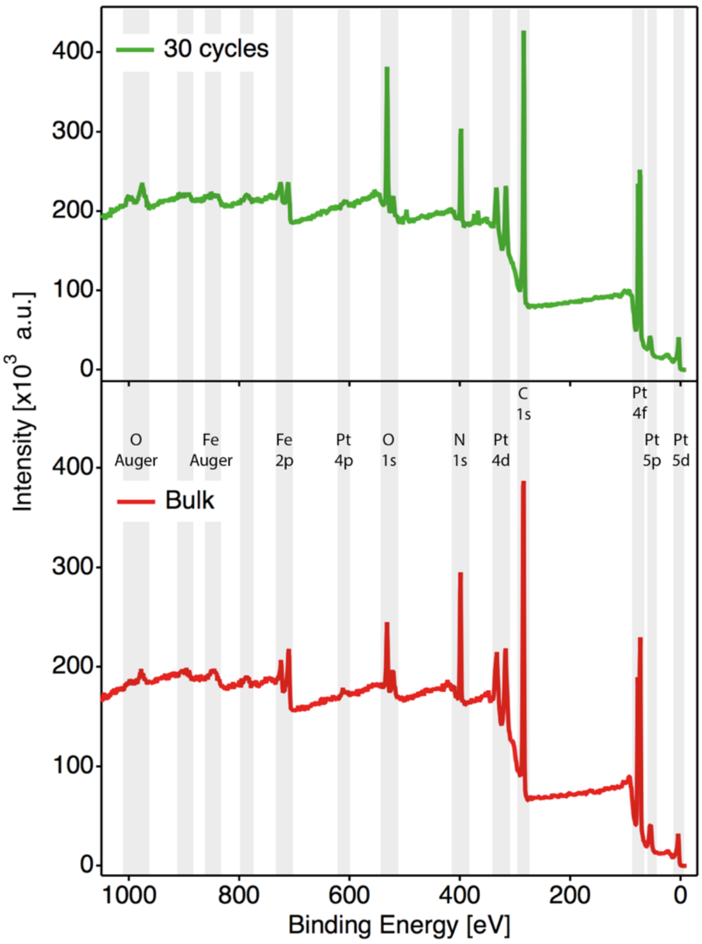

Figure S6. 2D-GIXRD data processing consisted of transforming the real space image (a) into a Q-space image (b). Then 1D XRD profiles were extracted by simple sector integration of the masked Q-space image. Thus, in-plane (from 150º to 180º, c) and out-of-plane (from 60º to 120º, d) diffractograms were obtained.

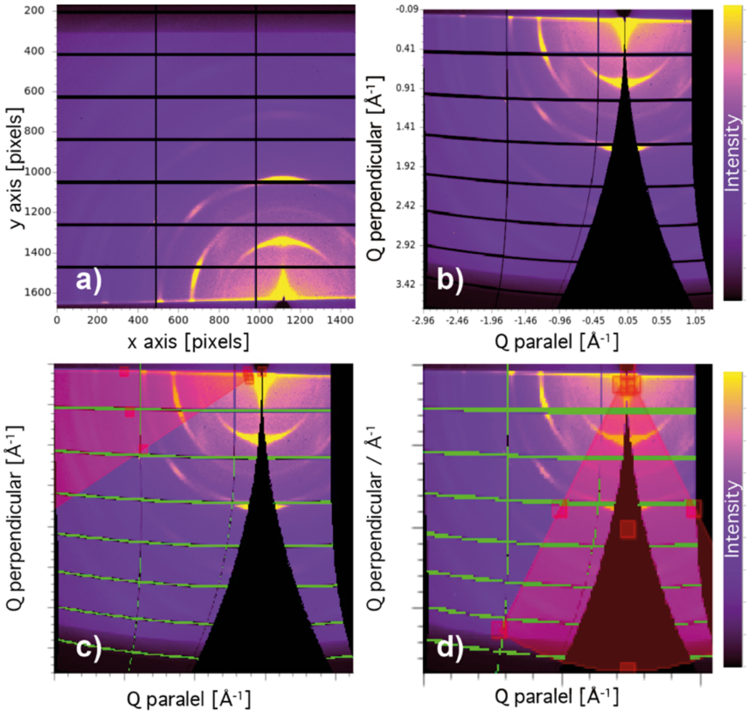

Figure S7. Raman spectra at 300 K (black) and 79 K (red) of a 90-cycles film onto Si/Ti (3 nm)/Au (15 nm). Spectra have been vertically shifted to better appreciate peaks position. Inset shows the normalized ν(CN) vibration around 2200 cm^{-1} which is highlighted in grey in the full spectra. A displacement of the peaks of 7.5 cm^{-1} can be observed with temperature. Moreover, a small shift towards higher frequencies is also measured in the ring stretching mode of the pyridine molecule around 1020 cm^{-1} and an enhancement is observed in the bending (∂) and stretching (ν) modes of the pyridine molecule at 1225 cm^{-1} and 1610 cm^{-1} in the LS state. The peak marked with (*) was found to be characteristic of a deterioration of the sample during measurement that could not be avoided due to the long exposition time required to record a spectrum with an acceptable signal-to-noise ratio.

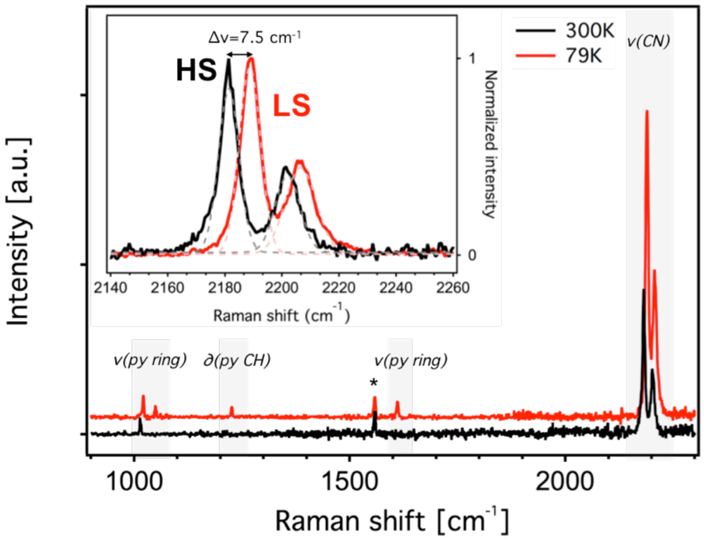

Figure S8. SCO transition in [Fe(py)$_2${Pt(CN)$_4$}] films as measured with SQUID magnetometry. a) Temperature-dependent magnetic susceptibility measurements of a Mylar/Ti/Au (15 nm)/[Fe(py)$_2${Pt(CN)$_4$}] 60 and 30 cycles samples.

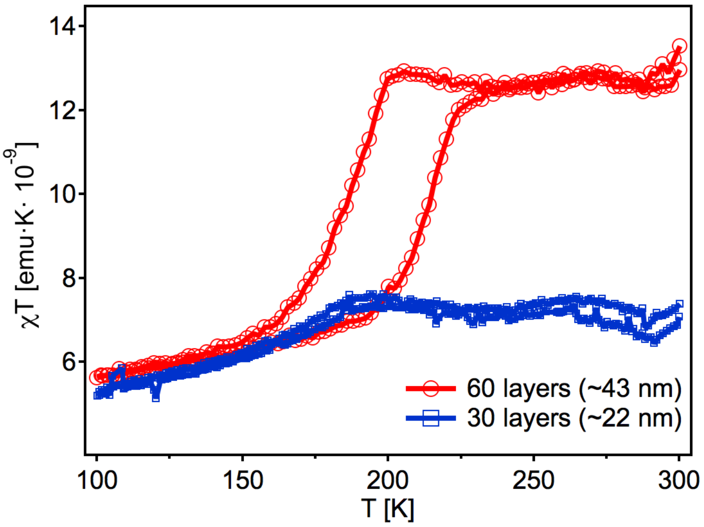

Figure S9. XAS spectra measured for a single crystal sample (Bulk-1) and a microcrystalline powder sample (Bulk-2) at 295, 100 and 4 K.

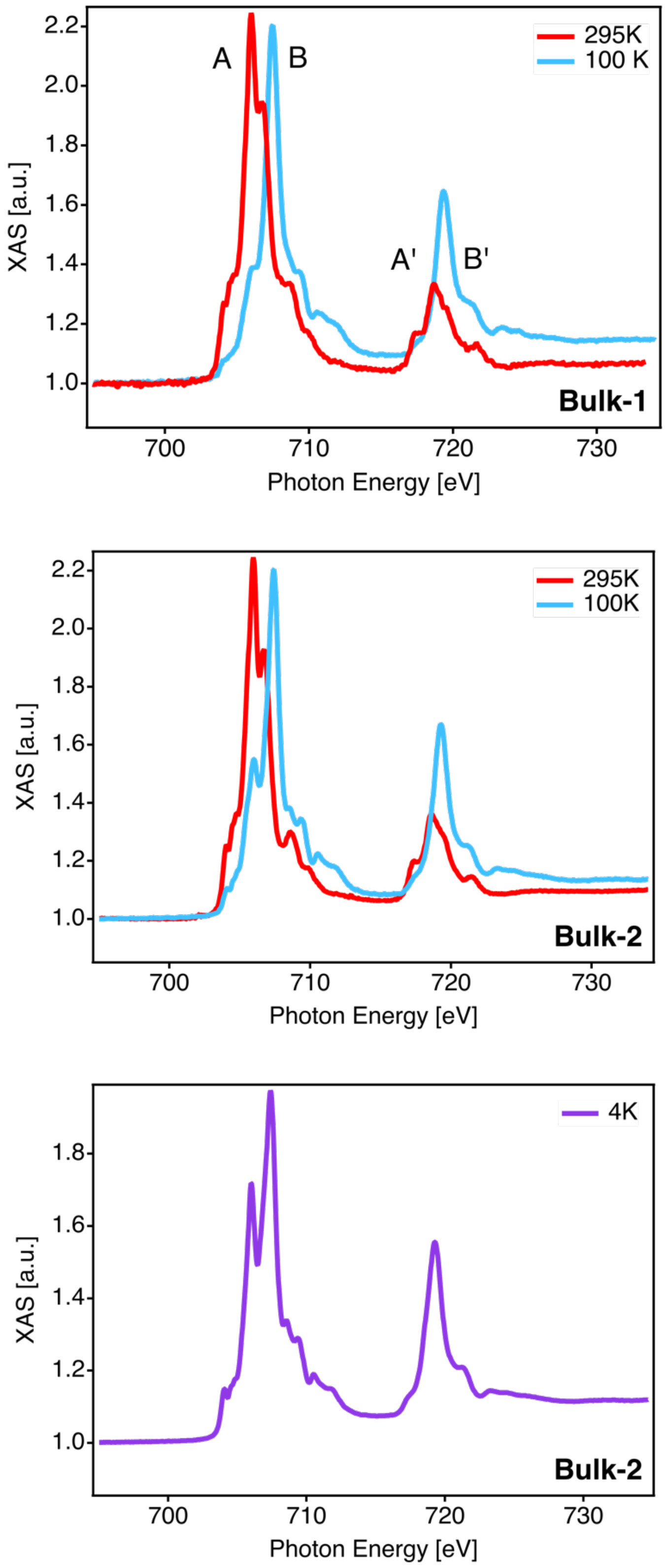

Figure S10. SCO behaviour of bulk $[Fe(py)_2\{Pt(CN)_4\}]$ samples with different crystallinity and particle size. From left to right: SEM images with their respective calculated particle size, PXRD diffractograms with the corresponding crystallite size as calculated using Scherrer's equation ([111] reflection) and the unit cell parameters that resulted from the LeBail profile fitting, and temperature-dependent magnetic susceptibility SQUID measurements with the calculated hysteresis (Hys) and transition temperatures ($T_{1/2}$). Scherrer's law was not applied to bulk-1, as this equation is only valid for a smaller crystallite size range.[8-10]

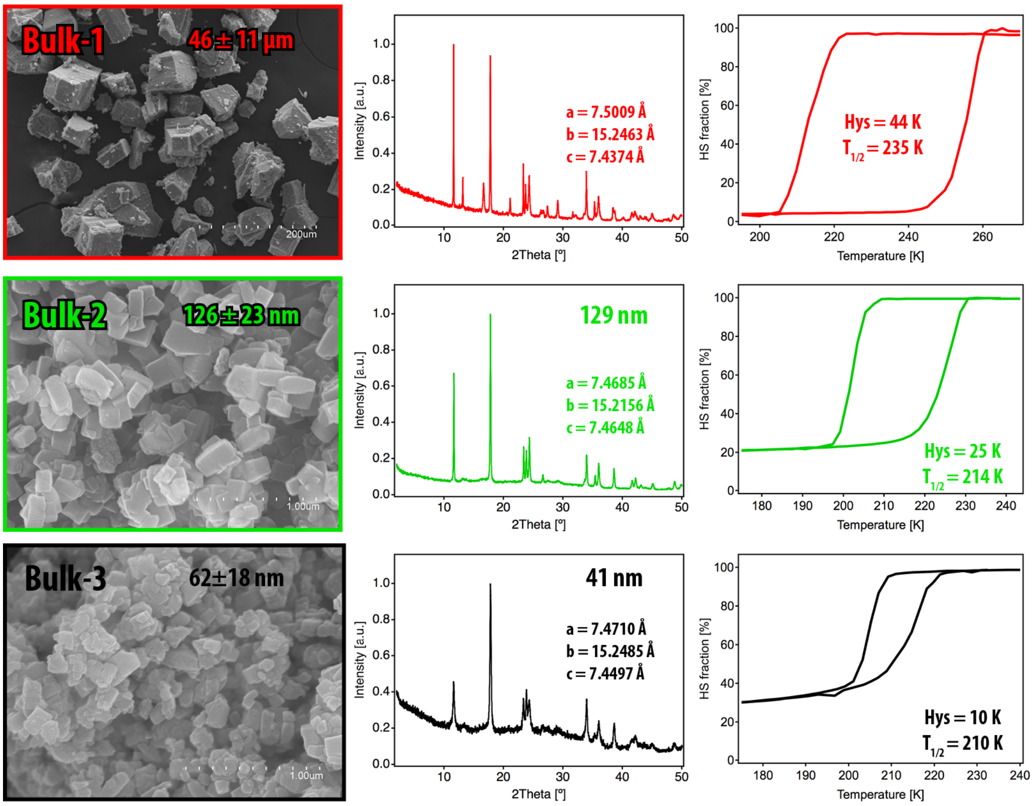

Figure S11. LeBail refinements of bulk $[Fe(py)_2\{Pt(CN)_4\}]$ samples.

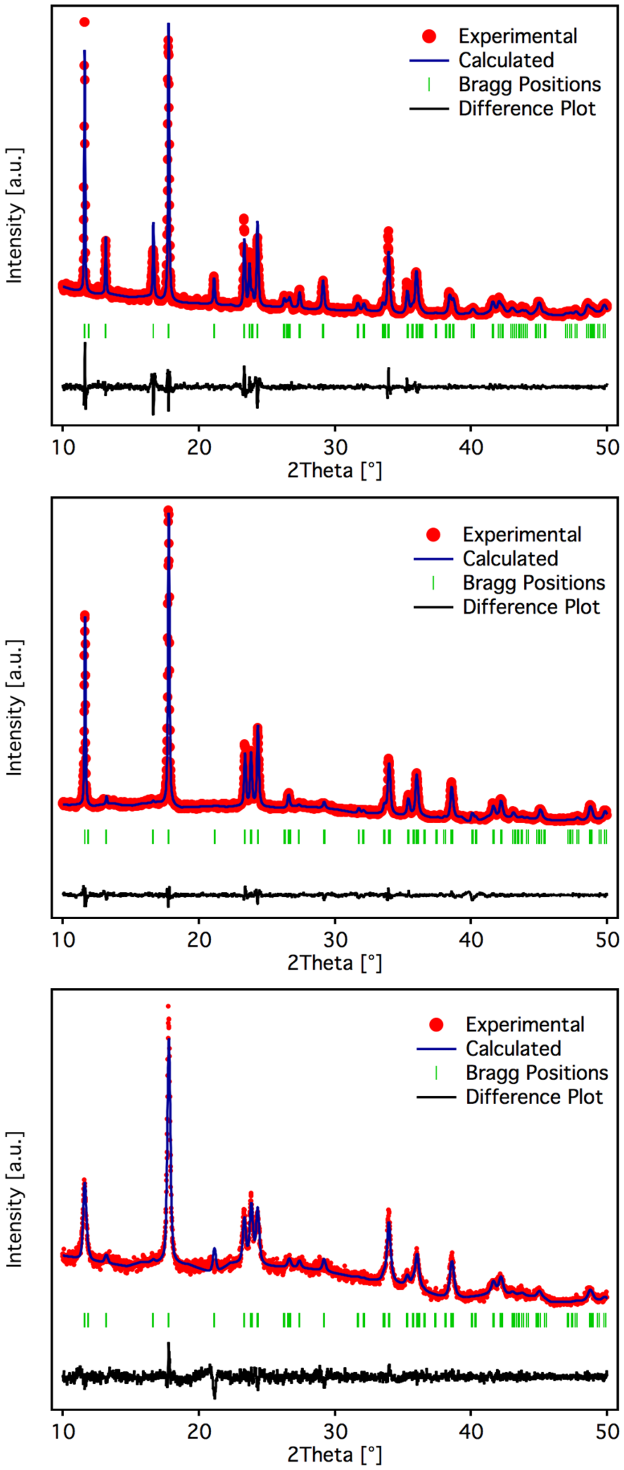

Table S1. Summary of the parameters obtained from the LeBail refinements.

	**a [Å]**	**b [Å]**	**c [Å]**	**V [$Å^3$]**	**R_e [%]**	**R_p [%]**	**R_{wp} [%]**	**gof**
**Bulk-1**	7.5009	15.2463	7.4374	850.55	6.53	7.53	10.1	1.5
**Bulk-2**	7.4685	15.2156	7.4648	848.28	4.54	4.63	6.34	1.4
**Bulk-3**	7.4710	15.2485	7.4497	848.68	4.78	4.68	6.08	1.3

Figure S12. 7-cycles sample XAS spectra at 295 K collected at the beginning(end) of the cooling down(up) cycles.

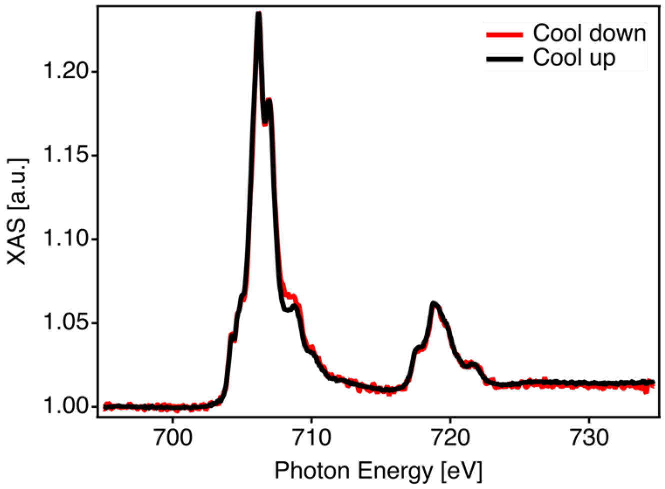

Figure S13. 7-cycles sample XAS spectra at 295, 175 and 100 K (dark dots) and corresponding fitted spectra (light line). Spectra have been shifted for clarity.

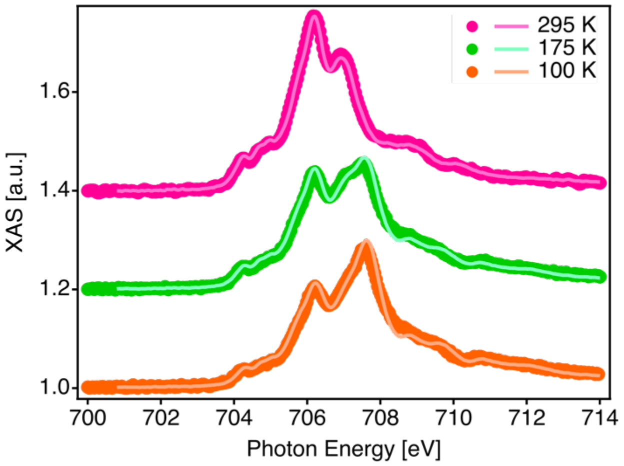

Figure S14. XAS spectra at 100 K (dark dots) and corresponding fitted spectra (light line) as function of the number of growth cycles.

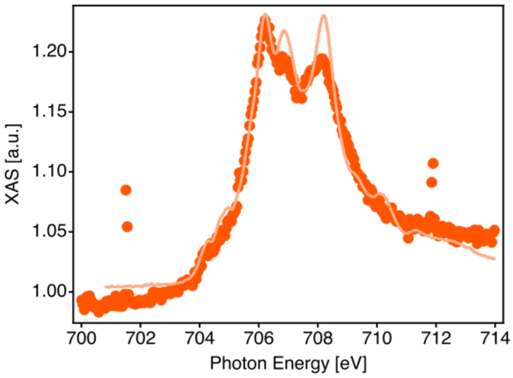

2 cycles

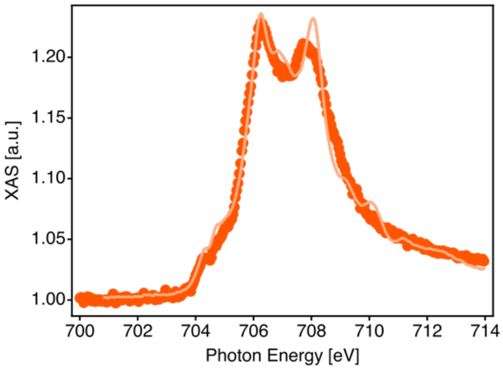

3 cycles

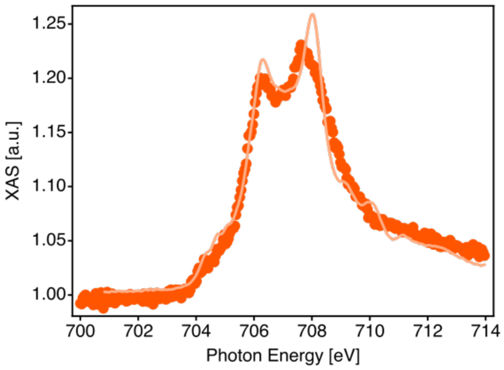

5 cycles

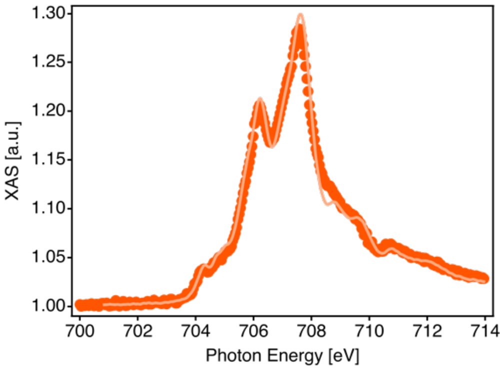

7 cycles

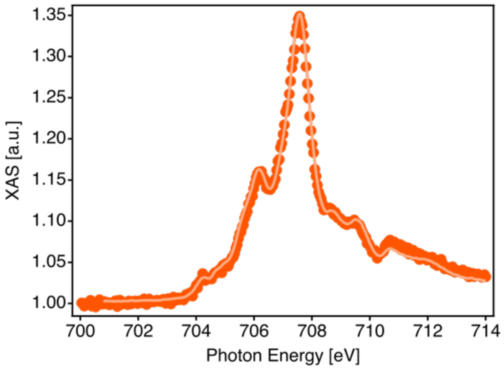

15 cycles

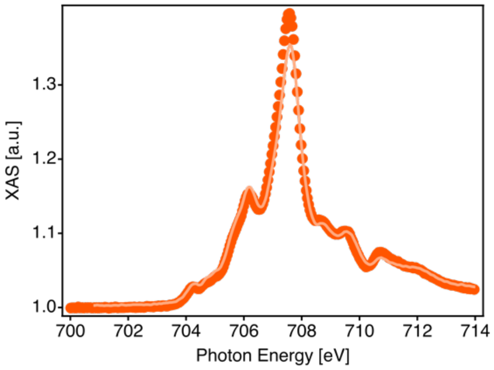

30 cycles

Figure S15. 1 x 1 μm^2 AFM topography, Prewitt edge and phase images of 2 (a), 5 (b), 7 (c), 10 (d) and 15 (e) cycles samples.

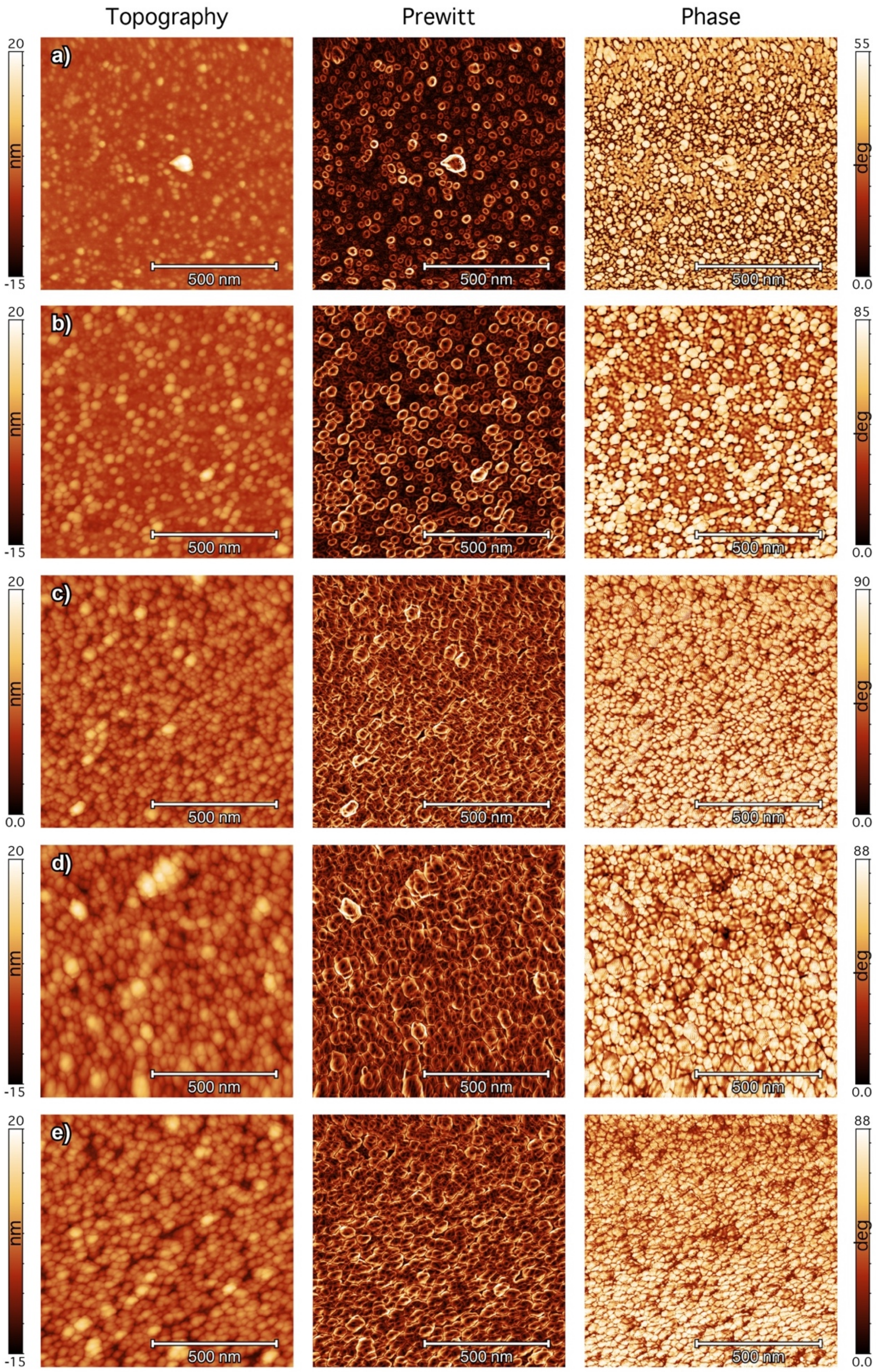

Figure S16. Evolution of mean lateral grain size and grade of coalescence with the number of cycles. Values were calculated by analysing AFM topography images with the Gwyddion software package.[11]

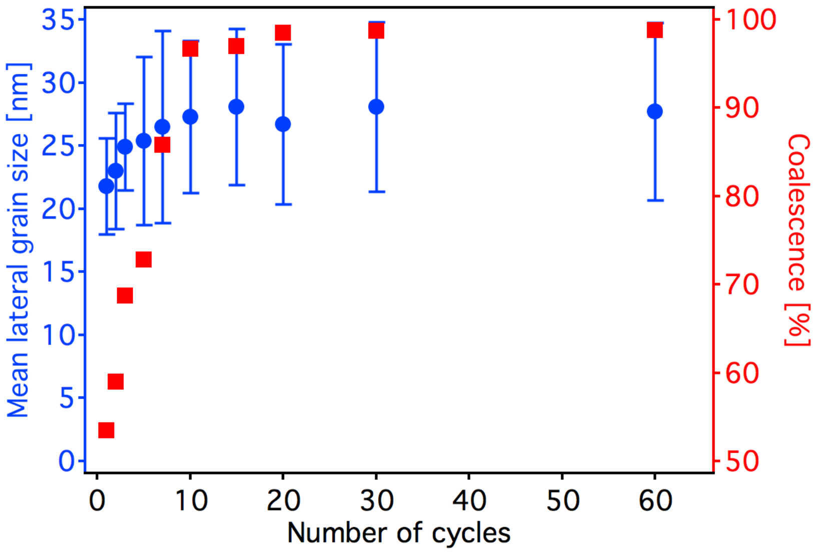

Figure S17. Evolution of RMS roughness with the number of cycles, which shows an exponential-like increase from ~0.8 nm for 1 cycle to ~5.5 nm after 20 cycles.

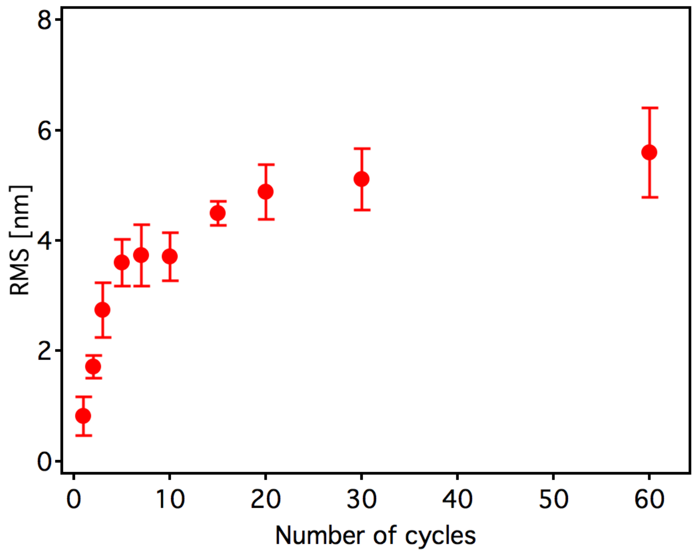

Table S2. Summary of the film microstructure parameters obtained by analysing AFM images with the Gwyddion software package.[8] Mean grain height refers to peak-to-valley height values extracted from height profiles of AFM topography images.

Number of cycles	Thickness [nm]	Mean grain height [nm]	Mean lateral grain size [nm]	Coalescence [%]	RMS [nm]
**1**	-	0.7 ± 0.2	21.8 ± 3.8	53.5	0.8 ± 0.3
**2**	-	1.4 ± 0.4	23 ± 4.6	59.0	1.7 ± 0.2
**3**	-	2.3 ± 0.7	24.9 ± 3.4	68.7	2.7 ± 0.5
**5**	-	3.1 ± 0.9	25.4 ± 6.6	72.8	3.6 ± 0.4
**7**	-	3.2 ± 0.7	26.5 ± 7.6	85.8	3.7 ± 0.6
**10**	7.8 ± 1.2	3.0 ± 1.0	27.3 ± 6.0	96.7	3.7 ± 0.4
**15**	12.2 ± 1.9	3.0 ± 1.1	28.1 ± 6.2	97.0	4.5 ± 0.2
**20**	14.9 ± 1.3	3.7 ± 1.5	26.7 ± 6.4	98.5	4.8 ± 0.5
**30**	22.4 ± 3.0	3.5 ± 1.1	28.1 ± 6.7	98.7	5.1 ± 0.6
**60**	42.5 ± 4.5	3.3 ± 0.4	27.7 ± 7.0	98.8	5.6 ± 0.8

Figure S18. Device fabrication. a) Optical image of Au (50 nm)/[Fe(py)$_2${Pt(CN)$_4$}] (90 layers)/Au (70 nm) vertical cross junctions fabricated over a Si/SiO$_2$ (285 nm) wafer with a diameter of 2". First, bottom Au electrodes were deposited by shadow mask on the Si/SiO$_2$ wafer using a Createc MBE evaporator with a base pressure of 10^{-9} mbar. Then, wafer was removed from the evaporator and a 90 layer [Fe(py)$_2${Pt(CN)$_4$}] film was grown by sequential immersion over the Au electrodes previously functionalized as described in the main text. Finally, wafer was transferred again inside the evaporator and top Au electrode was evaporated by shadow mask to fabricate a cross junction configuration. As it can be observed in the picture, more than 80 cross junctions were fabricated at the same time with cross sections of: 50 x 50 μm^2, 100 x 100 μm^2 and 500 x 500 μm^2. b) IV curve measured on a cross junction with area of 50 x 50 μm^2 of sample shown in (a). A probe station in a 2-points configuration was used to test all the fabricated junctions. A bias voltage was applied using a Yokogawa GS200 voltage source and current through the junction was measure using a Keithley 6517B electrometer. All the junctions fabricated presented a short-circuited behaviour as the one shown in (b).

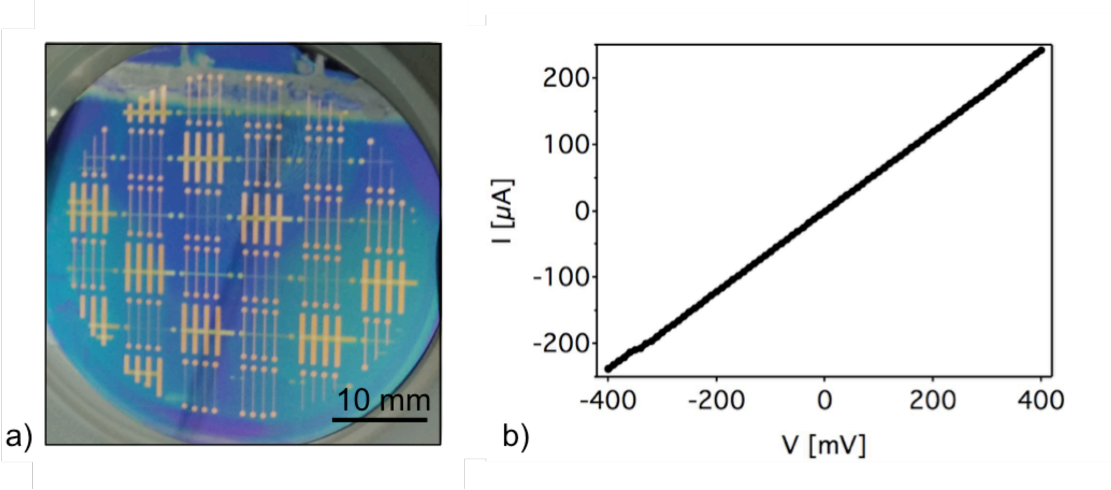

References:

1 T. Roisnel and J. Rodríguez-Carvajal, *Mater. Sci. Forum*, 2001, **378-381**, 118–123.
2 J. Filik, A. W. Ashton, Chang, P.C.Y., P. A. Chater, S. J. Day, M. Drakopoulos, M. W. Gerring, M. L. Hart, O. V. Magdysyuk, S. Michalik, A. Smith, C. C. Tang, N. J. Terrill, M. T. Wharmby and H. Wilhelm, *J. Appl. Cryst*, 2017, 969–966.
3 T. Kitazawa, Y. Gomi, M. Takahashi, M. Takeda, M. Enomoto, A. Miyazaki and T. Enoki, *J. Mater. Chem.*, 1996, **6**, 119–3.
4 T. Kitazawa, K. Hosoya, M. Takahashi, M. Takeda, I. Marchuk and S. M. Filipek, *J. Radioanal. Nucl. Chem.*, 2003, **255**, 509–512.
5 S. Sakaida, K. Otsubo, O. Sakata, C. Song, A. Fujiwara, M. Takata and H. Kitagawa, *Nat. Chem.*, 2016, 377–383.
6 P. Ohresser, E. Otero, F. Choueikani, K. Chen, S. Stanescu, F. Deschamps, T. Moreno, F. Polack, B. Lagarde, J. P. Daguerre, F. Marteau, F. Scheurer, L. Joly, J. P. Kappler, B. Muller, O. Bunau and P. Sainctavit, *Rev. Sci. Instrum.*, 2014, **85**, 013106–9.
7 Z. Lazic, V. Chamritski, D. Pooke, S. M. Valvidares, E. Pellegrin, S. Ferrer, X. Granados and X. Obradors, *J. Phys. Conf. Ser.*, 2013, **425**, 102003–5.
8 A. W. Burton, K. Ong, T. Rea and I. Y. Chan, *Microporous Mesoporous Mater.*, 2009, **117**, 75–90.
9 U. Holzwarth and N. Gibson, *Nat. Nanotech.*, 2011, **6**, 534–534.
10 K. He, N. Chen, C. Wang, L. Wei and J. Chen, *Cryst. Res. Technol.*, 2018, **53**, 1700157–6.
11 D. Nečas and P. Klapetek, *Cent. Eur. J. Phys.*, 2012, **10**, 181–188.
